# Regulation of Discrete Functional Responses by Syk and Src Family Tyrosine Kinases in Human Neutrophils

**DOI:** 10.1155/2017/4347121

**Published:** 2017-04-22

**Authors:** Thornin Ear, Olga Tatsiy, Frédérick L. Allard, Patrick P. McDonald

**Affiliations:** Pulmonary Division, Faculty of Medicine, Université de Sherbrooke and CRCHUS, Sherbrooke, QC, Canada

## Abstract

Neutrophils play a critical role in innate immunity and also influence adaptive immune responses. This occurs in good part through their production of inflammatory and immunomodulatory cytokines, in conjunction with their prolonged survival at inflamed foci. While a picture of the signaling machinery underlying these neutrophil responses is now emerging, much remains to be uncovered. In this study, we report that neutrophils constitutively express various Src family isoforms (STKs), as well as Syk, and that inhibition of these protein tyrosine kinases selectively hinders inflammatory cytokine generation by acting posttranscriptionally. Accordingly, STK or Syk inhibition decreases the phosphorylation of signaling intermediates (e.g., eIF-4E, S6K, and MNK1) involved in translational control. By contrast, delayed apoptosis appears to be independent of either STKs or Syk. Our data therefore significantly extend our understanding of which neutrophil responses are governed by STKs and Syk and pinpoint some signaling intermediates that are likely involved. In view of the foremost role of neutrophils in several chronic inflammatory conditions, our findings identify potential molecular targets that could be exploited for future therapeutic intervention.

## 1. Introduction

Neutrophils have long been known to play a critical role in host defense against infectious agents. However, the contribution of neutrophils to host immunity extends well beyond their traditional depiction as professional phagocytes specialized in pathogen clearance. It is now widely recognized that neutrophils and their products condition the onset and evolution of both innate immunity and the ensuing immune response [1, 2]. This occurs in good part through the generation of numerous proinflammatory mediators (chemokines, cytokines, and lipid mediators) by activated neutrophils [3], in conjunction with the increased persistence of neutrophils in inflamed tissues (relative to their circulating counterparts). We and the others have shown that the production of inflammatory cytokines by neutrophils, as well as their delayed apoptosis, are controlled by discrete signaling cascades, including the TAK1, IKK, p38 MAPK, MEK/ERK, and PI3K pathways [4–11]. We further showed that these signaling pathways control cytokine production through downstream effectors such as NF-*κ*B, CREB, and C/EBP transcription factors [8, 12–14]. We also showed that the above signaling cascades affect cytokine generation and delayed apoptosis translationally [8, 10, 15]. Thus, a picture is gradually emerging, of how various kinases and their downstream targets govern key neutrophil responses.

Whether other kinases also participate in controlling the aforementioned neutrophil responses remains to be explored. In this regard, one of the most immediate events occurring following neutrophil activation is the phosphorylation of cellular proteins by nonreceptor tyrosine kinases such as members of the Src family (including Hck, Fgr, and Lyn), as well as Syk [16–22]. Accordingly, the Src family of protein tyrosine kinases (STKs) and Syk have been reported to couple Fc receptors, adhesion receptors (integrins and selectins), and chemoattractant receptors to several classical effector functions of neutrophils, including phagocytosis, degranulation, ROS production, and leukotriene synthesis, though these tyrosine kinases seem to affect neutrophil migration to a lesser extent [23–25]. In contrast, a role for Src family and Syk tyrosine kinases in controlling inflammatory cytokine production or delayed apoptosis has yet to be described in human neutrophils.

We now report that inhibition of STKs or Syk selectively hinders inflammatory cytokine generation by acting posttranscriptionally. Accordingly, STK or Syk inhibition decreases the phosphorylation of signaling intermediates (e.g., eIF4B, S6K, and MNK1) involved in translational control. By contrast, delayed apoptosis appeared to be independent of either STKs or Syk. Our data therefore significantly extend our understanding of which neutrophil responses are governed by STKs and Syk and pinpoint some of the likely mechanisms involved.

## 2. Materials and Methods

### 2.1. Antibodies and Reagents

Antibodies raised against STK isoforms and *β*-actin were from Santa Cruz Biotechnology (Santa Cruz, CA, USA); antibodies against Syk, as well as all phospho antibodies, were from Cell Signaling (Beverly, MA, USA). Ficoll-Paque Plus was from GE Biosciences (Baie-d'Urfé, QC, Canada); endotoxin-free (<2 pg/ml) RPMI 1640 was from Wisent (St-Bruno, QC, Canada). Recombinant human cytokines were from R&D Systems (Minneapolis, MN, USA), and UltraPure LPS (from *E. coli* 0111:B4) was from InvivoGen (San Diego, CA, USA). Dimethyl sulfoxide (DMSO), N-formyl-methionyl-phenylalanine (fMLP), and phenylmethanesulphonyl fluoride (PMSF) were from Sigma-Aldrich (St. Louis, MO, USA). Diisopropyl fluorophosphate (DFP) was from Bioshop Inc. (Burlington, ON, Canada). The protease inhibitors, aprotinin, 4-(2-aminoethyl) benzenesulfonyl fluoride (AEBSF), leupeptin, and pepstatin A, were all from Roche (Laval, QC, Canada). Kinase inhibitors were all purchased through Cedarlane Labs (Mississauga, Canada). All other reagents were of the highest available grade, and all buffers and solutions were prepared using pyrogen-free clinical grade water.

### 2.2. Cell Isolation and Culture

Neutrophils were isolated from the peripheral blood of healthy donors, following a protocol that was duly approved by an institutional ethics committee. The entire procedure was carried out at room temperature and under endotoxin-free conditions, as described previously [26]. Purified neutrophils were resuspended in RPMI 1640 supplemented with 5% autologous serum, at a final concentration of 5 × 10^6^ cells/ml (unless otherwise stated). As determined by Wright staining and FACS analysis, the final neutrophil suspensions contained less than 0.1% monocytes or lymphocytes; neutrophil viability exceeded 98% after up to 4 h in culture, as determined by trypan blue exclusion and by annexin V/propidium iodide FACS analysis.

### 2.3. Immunoblots

Cells were incubated at 37°C in the presence or absence of stimuli. Incubations were stopped by adding equivalent volumes of ice-cold PBS supplemented with DFP (2 mM, final concentration) and phosphatase inhibitors (10 mM NaF, 1 mM Na_3_VO_4_, and 10 mM Na_4_P_2_O_7_). For whole-cell samples, boiling 2X sample buffer was added directly to cell pellets, which were briefly vortexed and placed in boiling water for a further 5 min. Samples thus prepared were sonicated to disrupt chromatin and stored at −20°C prior to analysis. For subcellular fractions, cells were resuspended in relaxation buffer prior to disruption by nitrogen cavitation, as described previously [27, 28]. Denatured samples were electrophoresed, transferred onto nitrocellulose, and processed for immunoblot analysis as previously described [27].

### 2.4. Real-Time PCR Analyses

Procedures and primers used are exactly as described before [13].

### 2.5. ELISA Analyses

Neutrophils were cultured in 24-well plates at 37°C under a 5% CO_2_ atmosphere, in the presence or absence of stimuli and/or inhibitors, for the indicated times. Culture supernatants were carefully collected, snap-frozen in liquid nitrogen, and stored at −80°C. Samples were analyzed in ELISA using commercially available capture and detection antibody pairs (R&D Systems, BD Biosciences).

### 2.6. Determination of Neutrophil Apoptosis

After the desired culture period, neutrophils (5 × 10^5^ cells) were washed twice in ice-cold PBS containing 5 mM EDTA, then once more in cold PBS, and incubated on ice for 15 min with FITC-conjugated annexin V. Cells were then counterstained with propidium iodide and analyzed (minimum of 10,000 cells) on a Cytoflex instrument (Beckman Coulter) using the CytExpert software.

### 2.7. Data Analysis

All data are represented as the mean ± SEM of at least three independent experiments. Statistical differences were analyzed by Student's *t*-test for paired data, using Prism 7 software (GraphPad Software, San Diego, CA, USA).

## 3. Results

### 3.1. Expression and Distribution of Src Family and Syk Tyrosine Kinases in Human Neutrophils

**Figure 1:**
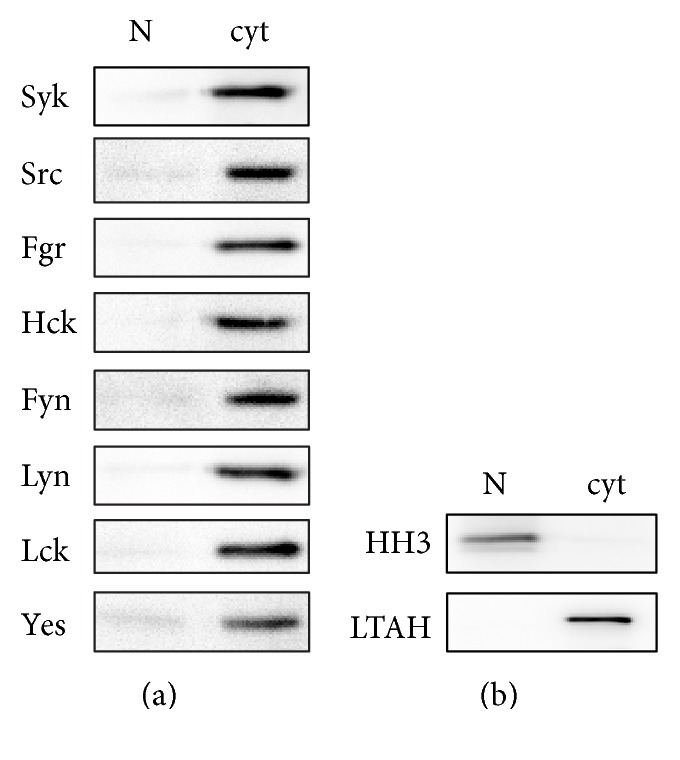
Expression and cellular distribution of Src family and Syk tyrosine kinases in human neutrophils. (a) Neutrophils (pmn) were disrupted by nitrogen cavitation; cytoplasmic and nuclear fractions were then processed for immunoblot analysis of Src family and Syk kinases (0.5 × 10^6^ cell equivalents were loaded per lane). (b) Neutrophil subcellular fractions prepared as above were processed for immunoblot analysis of the cytosolic marker leukotriene A4 hydrolase (LTAH) or the nuclear marker histone H3 (HH3). The experiment shown in this figure is a representative of three.

Previous studies have established that at least three STK isoforms are present in neutrophils (e.g., Fgr, Hck, and Lyn), as well as Syk [18–22]. To gain a more complete understanding of which isoforms are present (and where they localize), resting neutrophils were disrupted by nitrogen cavitation, and cytoplasmic and nuclear fractions were processed for immunoblot analysis.  Figure 1(a) shows that all STKs investigated, as well as Syk, are strictly cytoplasmic in human neutrophils. The purity of our subcellular fractions was ascertained for the presence of cytosolic and nuclear markers. As shown in Figure 1(b), strictly cytosolic proteins, such as leukotriene A4 hydrolase [29], were only detected in cytoplasmic fractions; conversely, histone H3 was exclusively nuclear, as expected.

### 3.2. Effect of STK and Syk Inhibition on Cytokine Expression and Release in Human Neutrophils

**Figure 2:**
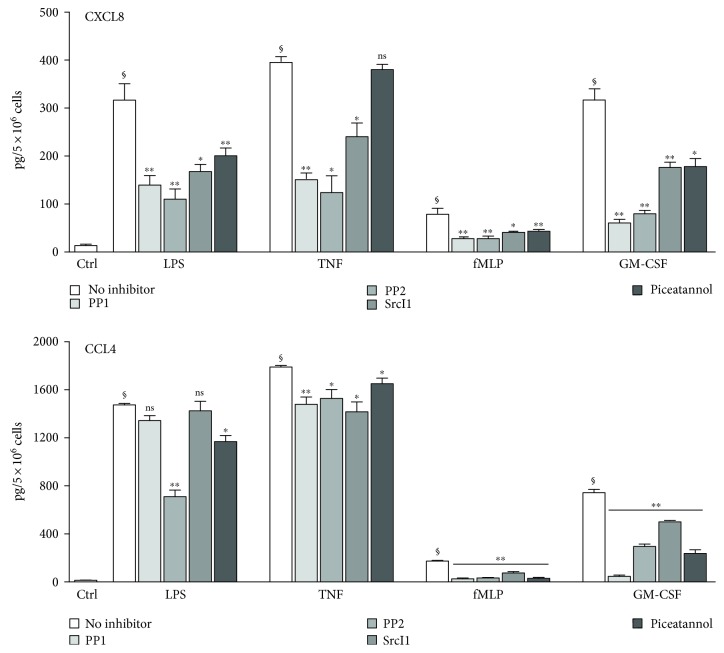
Effect of STK and Syk inhibition on inflammatory cytokine generation in human neutrophils. Cells were pretreated for 30 min in the absence or presence of STK inhibitors (10 *μ*M of PP1, PP2, or Srcl1) or of a Syk inhibitor (10 *μ*M piceatannol), prior to stimulation for 6 h with 1 *μ*g/ml LPS, 100 U/ml TNF*α*, 30 nM fMLP, or 1 nM GM-CSF. Culture supernatants were analyzed in ELISA. Results are expressed as mean ± SEM from at least 3 independent experiments, each performed in duplicate. §, *p* < 0.001 versus unstimulated cells (“ctrl”); ^∗^*p* < 0.05 versus matched condition without inhibitor; ^∗∗^*p* < 0.01 versus matched condition without inhibitor.

Whereas Src family and Syk tyrosine kinases play an important role in various classical functions of neutrophils, such as phagocytosis, ROS production, and leukotriene synthesis [23], the potential involvement of these kinases in cytokine generation has not been studied to date. To address this issue, neutrophils were pretreated with Src family inhibitors (i.e., PP1, PP2, and SrcI1) or a widely used Syk inhibitor (piceatannol), prior to stimulation with physiological stimuli; cytokine production was then assessed by ELISA. As shown in Figure 2, all inhibitors strongly repressed the inducible secretion of CXCL8 in response to LPS, TNF*α*, fMLP, or GM-CSF, with the notable exception of TNF-elicited CXCL8 release, which was consistently unaffected by Syk inhibition. The release of another chemokine, CCL4, was likewise hindered by all inhibitors tested in fMLP- and GM-CSF-stimulated cells; in LPS- or TNF-stimulated neutrophils, however, CCL4 release was only modestly affected by Syk or STK inhibitors (even though the inhibition was often statistically significant).

**Figure 3:**
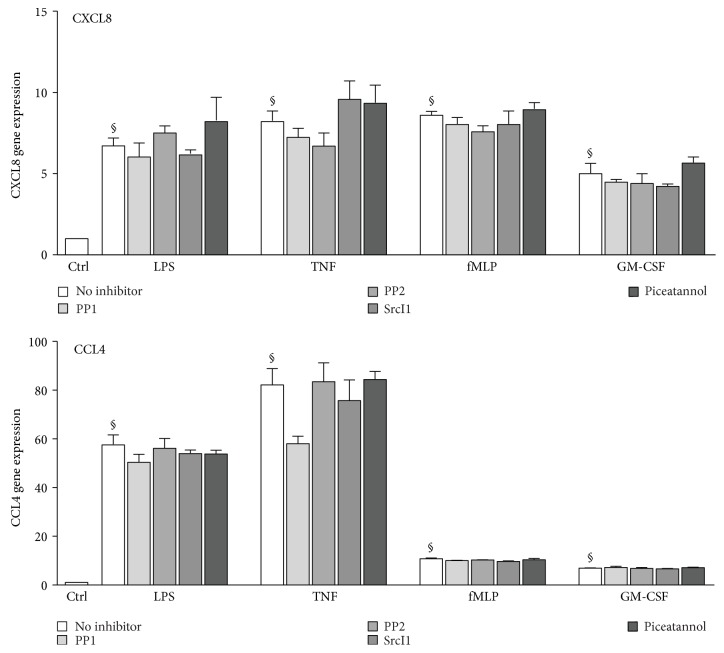
Effect of STK and Syk inhibition on inflammatory cytokine gene expression in human neutrophils. Cells were pretreated with STK or Syk inhibitors, prior to a 30 min stimulation with LPS, TNF*α*, fMLP, or GM-CSF, as described for Figure 2. Total RNA was isolated, reverse-transcribed, and analyzed for cytokine gene expression by real-time qPCR. Values were normalized over RPL32 and are represented as fold increase relative to unstimulated cells. Mean ± SEM from at least 3 independent experiments, each performed in duplicate. §, *p* < 0.007 versus unstimulated cells (“ctrl”).

Because inflammatory chemokine generation is typically preceded by an accumulation of the corresponding transcripts in neutrophils, we also investigated whether inhibition of Src family and Syk kinases would yield similar outcomes. In contrast to cytokine release, STK and Syk inhibition failed to significantly alter CXCL8 or CCL4 gene expression induced by LPS, TNF*α*, fMLP, or GM-CSF (Figure 3). Taken together, the above data indicate that in response to physiological agonists, STKs and Syk affect cytokine generation posttranscriptionally.

### 3.3. Translational Targets of STKs and Syk in Human Neutrophils

**Figure 4:**
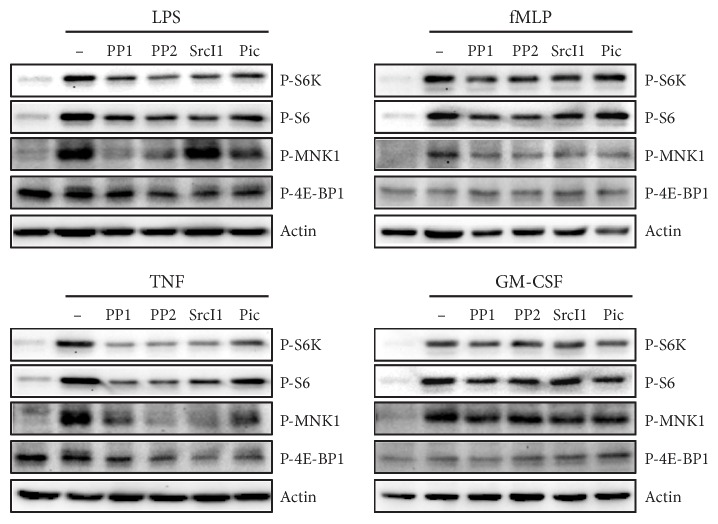
Potential translational targets of Syk and Src family tyrosine kinases in human neutrophils. Cells were pretreated with STK or Syk inhibitors, prior to a 10 min stimulation with LPS, TNF*α*, fMLP, or GM-CSF, as described for Figure 2. Samples were then processed for immunoblot analysis; membranes were also blotted for *β*-actin (as a loading control). Data are representative of at least 3 independent experiments.

We previously identified several signaling intermediates involved in the translational control of inflammatory cytokine production in neutrophils [8, 10, 15]. Since our present data indicates that STKs and Syk act posttranscriptionally towards this response, we investigated whether these tyrosine kinases might affect some of the translational events, which we identified in previous studies. As shown in Figure 4 and Figure S1 available online at https://doi.org/10.1155/2017/4347121, neutrophil pretreatment with STK or Syk inhibitors clearly diminished the LPS- or TNF-elicited phosphorylation of MNK1; of ribosomal S6 kinase and its substrate, the S6 ribosomal protein; and to a lesser extent, of 4E-BP1. A similar inhibition profile was observed in neutrophils activated with fMLP or GM-CSF, though the extent of inhibition was less pronounced than in LPS- or TNF-treated cells. Thus, Src and Syk kinases have the potential to modulate the translation of inflammatory cytokines by controlling discrete signaling intermediates.

### 3.4. Impact of STKs and Syk Kinases on Delayed Apoptosis in Human Neutrophils

**Figure 5:**
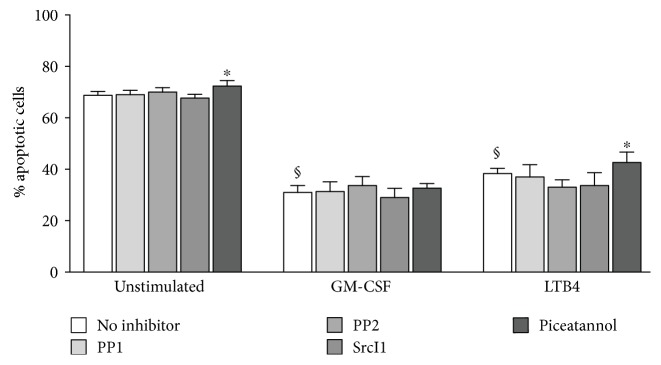
Effect of STK and Syk inhibition on delayed apoptosis in human neutrophils. Cells were pretreated with STK or Syk inhibitors, prior to a 20 h culture with LPS, TNF*α*, fMLP, or GM-CSF, as described for Figure 2. Cells were then processed for FACS analysis of annexin V binding; a minimum of 10,000 cells were processed for each sample. Results are expressed as mean ± SEM of at least 3 independent experiments. §, *p* < 0.001 versus unstimulated cells (“ctrl”); ^∗^*p* < 0.05 versus matched condition without inhibitor.

We finally determined whether Src family and Syk kinases might contribute to the apoptosis-delaying effect of GM-CSF or LTB4 in neutrophils. The reason for using LTB4 as a chemoattractant is that fMLP does not retard constitutive apoptosis in neutrophils. Neutrophils were pretreated with inhibitors of STKs or Syk and cultured overnight in the presence of GM-CSF or LTB4 before apoptosis assessment. As shown in Figure 5, piceatannol by itself exerted a small effect on spontaneous apoptosis (*p* = 0.04), whereas both GM-CSF and LTB4 potently countered the spontaneous apoptotic rate, as expected (Figure 5). In stimulated neutrophils, the STK inhibitors failed to affect the prosurvival effect of either GM-CSF or LTB4, whereas piceatannol slightly affected that of LTB4 (*p* = 0.04) but not that of GM-CSF (Figure 5). Thus, STKs or Syk exerts little or no effect on the delayed apoptotic response of neutrophils to growth factors or chemoattractants.

## 4. Discussion

A large number of inflammatory and immune processes have been reported to be influenced by neutrophils and their products in vivo, whence the sustained interest in the underlying signaling events. Following neutrophil exposure to various stimuli, protein tyrosine kinases such as STKs and Syk rapidly become activated and influence several classical functions of these cells, including phagocytosis, degranulation, ROS production, and leukotriene synthesis [23]. In this report, we show that STKs and Syk also participate in the control of another major functional response of neutrophils, that is, inflammatory cytokine generation, whereas they have little or no effect on either spontaneous or delayed apoptosis in these cells.

We first found that STK or Syk inhibition strongly represses the secretion of CXCL8 elicited by physiological neutrophil agonists (i.e., LPS, TNF*α*, fMLP, and GM-CSF), with the exception of TNF-induced CXCL8 release, which appeared to occur independently of Syk. Similarly, CCL4 secretion was hindered by STK and Syk inhibitors in fMLP- and GM-CSF-activated neutrophils but was only moderately unaffected by the inhibitors in LPS- or TNF-stimulated cells. Thus, depending on the cytokine and the stimulus, inflammatory cytokine generation can be controlled by STKs or Syk in neutrophils. These tyrosine kinases appeared to act posttranscriptionally, since the induction of inflammatory cytokine gene expression was unaffected by STK or Syk inhibition. Accordingly, several signaling intermediates known to affect cytokine translation were found to be under the control of STKs and Syk. Among these downstream targets, MNK1 is particularly relevant, as we recently showed it to participate in the translational regulation of cytokine generation in neutrophils [15]. As observed for MNK1, the inducible phosphorylation of the S6 kinase (and of its substrate, the S6 ribosomal protein) and to a lesser extent the inducible hyperphosphorylation of 4E-BP1 were impaired following STK or Syk inhibition. This again points towards translational control, given the known involvement of S6K, S6, and 4E-BP1 in cap-dependent protein translation in various cell types [30, 31]. Thus, it appears that STKs and Syk control inflammatory cytokine generation translationally in primary neutrophils.

In contrast to cytokine production, STKs and Syk affect other neutrophil responses only moderately, as in the case of chemotaxis [23–25], or not at all, as we found here in the case of constitutive and delayed apoptosis. In the latter instance, this was somewhat surprising, given that cytokine generation and delayed apoptosis have been shown to share many upstream signaling events in neutrophils. Our results therefore identify some of the differences in how these key neutrophil responses are governed.

Collectively, our data significantly extends our understanding of which neutrophil responses are governed by STKs and Syk (and which are not). More importantly, we uncovered some of the likely mechanisms involved in the translational control of cytokine generation by STKs and Syk. In view of the foremost role of neutrophils in several chronic inflammatory conditions, our findings identify potential molecular targets that could be exploited for future therapeutic intervention.

## Supplementary Material

Figure S1. Densitometric analyses of Figure 4 data. §, p≤0.01 vs unstimulated cells; ∗, p<0.05 vs matched condition without inhibitor; ∗∗, p<0.01 vs matched condition without inhibitor.
